# COST ANALYSIS OF THE ADULT DAY SERVICES PLUS TRIAL

**DOI:** 10.1093/geroni/igad104.0525

**Published:** 2023-12-21

**Authors:** Laura Pizzi, Katherine Prioli, Eric Jutkowitz, Alexa Molinari, Laura Gitlin

**Affiliations:** ISPOR - The Professional Society for Health Economics and Outcomes Research, Lawrenceville, New Jersey, United States; Rutgers University, Piscataway, New Jersey, United States; Brown University, Providence, Rhode Island, United States; Rutgers University, Picataway, New Jersey, United States; Drexel University, Philadelphia, Pennsylvania, United States

## Abstract

We assessed costs associated with ADS Plus vs. control from payer and societal perspectives using trial data. Costs were valued in $US 2021 and included ADS Plus program delivery, direct healthcare utilization (HCU), formal care and social services (FCSS), and indirect caregiver time (CGT). Payer perspective analyses included HCU and FCSS costs, with and without ADS Plus program delivery costs; societal perspective included these plus CGT costs. Analysis consisted of the mean per-dyad difference in change for ADS Plus (n=102) vs. control (n=101) at 6 and 12-months. ADS Plus program delivery costs were $671. At 6-months, comparing ADS Plus to control, there were mean savings in HCU ($79), FCSS ($1,038), and CGT ($6,426). Payer perspective total savings for ADS Plus were $446 and $1,117 with and without ADS Plus program delivery respectively; societal perspective savings were $6,872 and $7,543, with vs. without AD Plus program delivery, respectively. At 12-months for ADS Plus vs. control, HCU savings were $1,686 and FCSS savings were $290. CGT was $76 higher for ADS Plus. Payer perspective savings were $1,305 and $1,976 with and without ADS Plus program delivery respectively; societal perspective savings were $1,228 with ADS Plus delivery and $1,899 without it. ADS Plus costs were lower than control thus representing potential savings from both the payer and societal perspectives. Savings were primarily driven by lower costs in healthcare utilization including inpatient hospitalizations, outpatient visits, and home health aide services. Though favorable, findings were not statistically significant and should be interpreted with caution.

